# Medical Costs of Substance Use Disorders in the US Employer-Sponsored Insurance Population

**DOI:** 10.1001/jamanetworkopen.2022.52378

**Published:** 2023-01-24

**Authors:** Mengyao Li, Cora Peterson, Likang Xu, Christina A. Mikosz, Feijun Luo

**Affiliations:** 1 National Center for Injury Prevention and Control, US Centers for Disease Control and Prevention, Atlanta, Georgia; 2 National Center for Chronic Disease Prevention and Health Promotion, US Centers for Disease Control and Prevention, Atlanta, Georgia

## Abstract

**Importance:**

Direct costs of substance use disorders (SUDs) in the United States are incurred primarily among the working-age population. Quantifying the medical cost of SUDs in the employer-sponsored insurance (ESI) population can improve understanding of how SUDs are affecting workplaces and inform decision-making on the value of prevention strategies.

**Objective:**

To estimate the annual attributable medical cost of SUDs in the ESI population from the health care payer perspective.

**Design, Setting, and Participants:**

In this economic evaluation, Merative MarketScan 2018 databases were weighted to represent the non–Medicare eligible ESI population. Regression and mathematical modeling of medical expenditures controlled for insurance enrollee demographic, clinical, and insurance factors to compare enrollees with and without an SUD diagnosis to identify the annual attributable medical cost of SUDs. Data analysis was conducted from January to March 2022.

**Exposures:**

*International Statistical Classification of Diseases, Tenth Revision, Clinical Modification* SUD diagnoses on inpatient or outpatient medical records according to Clinical Classifications Software categories (alcohol-, cannabis-, hallucinogen-, inhalant-, opioid-, sedative-, stimulant-, and other substance-related disorders).

**Main Outcomes and Measures:**

Annual SUD medical cost in the ESI population overall and by substance type (eg, alcohol). Number of enrollees with an SUD diagnosis and the annual mean cost per affected enrollee of SUD diagnosis (any and by substance type) are also reported.

**Results:**

Among 162 million ESI enrollees, 2.3 million (1.4%) had an SUD diagnosis in 2018. The regression analysis sample included 210 225 individuals with an SUD diagnosis (121 357 [57.7%] male individuals; 68 325 [32.5%] aged 25-44 years) and 1 049 539 individuals with no SUD diagnosis. The mean annual medical cost attributable to SUD diagnosis per affected enrollee was $15 640 (95% CI, $15 340-$15 940), and the total annual medical cost in the ESI population was $35.3 billion (2018 USD). Alcohol use disorder ($10.2 billion) and opioid use disorder ($7.3 billion) were the most costly.

**Conclusions and Relevance:**

In this economic evaluation of medical expenditures in the ESI population, the per-person and total medical costs of SUDs were substantial. Strategies to support employees and their health insurance dependents to prevent and treat SUDs can be considered in terms of potentially offsetting the existing high medical cost of SUDs. Medical expenditures for SUDs represent the minimum direct cost that employers and health insurers face because not all people with SUDs have a diagnosis, and costs related to absenteeism, presenteeism, job retention, and mortality are not addressed.

## Key Points

**Question:**

What is the medical cost of substance use disorders (SUDs) for US employers, employees, and their health insurance payers?

**Findings:**

In this economic evaluation of 162 million non–Medicare eligible enrollees with employer-sponsored health insurance in 2018, 2.3 million had an SUD diagnosis. The annual attributable medical expenditure was $15 640 per affected enrollee and $35.3 billion in the population; alcohol-related disorders ($10.2 billion) and opioid-related disorders ($7.3 billion) were the most costly.

**Meaning:**

These findings suggest that employers and health insurance payers can support employees and their dependents to prevent and treat SUDs and consider prevention strategies in terms of potentially offsetting the existing high medical cost of SUDs.

## Teaser

This economic analysis estimates the annual attributable medical costs of substance use disorders in the employer-sponsored health insurance population from the health care payer perspective.

## Introduction

The direct costs of substance use disorders (SUDs) in the United States are incurred primarily among the working-age population. During 2001 to 2020, more than 90% of deaths (865 732 of 932 573) caused by drug or alcohol poisoning and nearly 80% of nonfatal poisoning emergency department visits (20.1 million of 25.7 million) occurred among people aged 20 to 64 years.^1,2^ Overall, 11% of US-employed adults (aged ≥18 years) reported having SUDs in 2020.^3^ More than half of employers with 50 or more employees surveyed in 2019 reported direct effects from the opioid overdose epidemic, such as impaired or decreased job performance.^4^ Previous estimates of SUD medical costs among US employees are well described but relied on workers’ self-reported health care utilization.^5^ Quantifying the medical cost of SUDs in the employer-sponsored insurance (ESI) population through analysis of actual financial transactions can highlight how SUDs affect workplaces and inform decision-making on the value of prevention strategies. This study aimed to estimate the annual attributable medical cost of SUDs in the ESI population from the health care payer perspective.

## Methods

This economic evaluation did not require evaluation by an institutional review board or patient informed consent because all data were publicly available and no human participants were involved (45 CFR part 46). This study followed Consolidated Health Economic Evaluation Reporting Standards (CHEERS) reporting guidelines for economic evaluations.^6^ Merative MarketScan 2018 Commercial Claims and Encounters Databases were weighted to represent the non–Medicare eligible (aged <65 years) US ESI population (active employees, early retirees, and COBRA continuees plus dependents; Medicare supplemental plans not included). The databases report expenditures for inpatient services, outpatient services, and outpatient drugs from approximately 350 health insurance payers (large employers, health plans, and government and public organizations). Race and ethnicity are not reported in this database. By comparing total annual medical expenditures among similar enrollees with and without SUD diagnoses (referred to as control participants), this analysis aimed to estimate the cost that could be eliminated through prevention or successful treatment of SUD (referred to as the attributable cost). The main outcome measure was the annual attributable SUD medical cost in the ESI population overall and by substance type (eg, alcohol). The number of enrollees with an SUD diagnosis in the ESI population and the annual mean attributable cost of SUD diagnosis (any and by substance type) per affected enrollee are also reported. SUD diagnosis was defined by *International Statistical Classification of Diseases, Tenth Revision, Clinical Modification* (*ICD-10-CM*) according to Clinical Classifications Software categories (alcohol-, cannabis-, hallucinogen-, inhalant-, opioid-, sedative-, stimulant-, and other substance-related disorders) on an inpatient or outpatient claim record (Table 1). Costs are reported in 2018 US dollars (unadjusted from data source); cost discounting was not applicable due to the 1-year analytic time horizon, which was selected to facilitate reporting in terms of annual cost, and the cost perspective was the health care payer.

**Table 1.** Substance Use Disorder Definition^a^

Substance	CCSR code	CCSR description	*ICD-10-CM* code
Alcohol	MBD017	Alcohol-related disorders	F10,^b^ G312, G621, I426, K292, K70, O354, O9931
Cannabis	MBD019, MBD030,^c^ MBD034^d^	Cannabis-related disorders	F12,^b^ T407^e^
Hallucinogen	MBD022, MBD031,^c^ MBD034^d^	Hallucinogen-related disorders	F16,^b^ T408,^e^ T409^e^
Inhalant	MBD023, MBD033,^c^ MBD034^d^	Inhalant-related disorders	F18,^b^ T410^e^
Opioid	MBD018, MBD028,^c^ MBD034^d^	Opioid-related disorders	F11,^b^ T400,^e^ T401, ^e^ T402,^e^ T403,^e^ T404,^e^ T406^e^
Sedative	MBD020, MBD032, MBD034^c^^,^^d^	Sedative-related disorders	F13,^b^ T426,^e^ T427^f^
Stimulant	MBD021, MBD029,^c^ MBD034^d^	Stimulant-related disorders	F14,^b^ F15,^b^ T405,^e^ T436^e^
Other	MBD025	Other specified substance-related disorders^g^	F19,^b^ O355, O9932

Abbreviations: CCSR, Clinical Classifications Software Refined; *ICD-10-CM*, *International Statistical Classification of Diseases, Tenth Revision, Clinical Modification*.

^a^ This table’s data are reproduced from the study by Peterson et al.^7^

^b^ This includes all codes in the series except for in remission (fifth digit of 1).

^c^ These were subsequent encounters.

^d^ Only sequela diagnoses from the listed substance (eg, stimulant) were included from CCSR MBD034.

^e^ Includes all codes in the series except for assault (sixth digit of 3) and underdosing (sixth digit of 6).

^f^ Includes all codes in the series except for assault (fifth digit of 3) and underdosing (fifth digit of 6).

^g^ Includes abuse or complications from other psychoactive substances and maternal care for drug use complicating pregnancy and childbirth.

### Statistical Analysis

Analysis was conducted with SAS version 9.4 (SAS Institute) and Stata version 17 (StataCorp) during January to March 2022. SUD diagnoses during the study period of January 1 to December 31, 2018, were assessed among all enrollees in the data source and weighted using data source methods to estimate the number of nationwide ESI enrollees with SUD diagnoses.^7^ Unweighted regression models used records from a subgroup of enrollees with complete data for analysis to compare enrollees with SUD diagnoses and control participants to identify the per-enrollee cost of SUDs within enrollees’ total medical expenditures. Regression models were unweighted because not all data relevant to estimate mean per-enrollee expenditures by health condition (eg, SUD) have available population weighting factors in the data source.^8^ Complete data per analyzed enrollee were continuous monthly insurance enrollment during the study period, mental health and SUD coverage, and nonmissing key data (sex, age, region of residence, health plan type [eg, preferred provider organization; health maintenance organization, and capitation plans excluded]), and 1 to 5 matched control enrollees. Exact matching criteria to select control enrollees were sex, age, region of residence, health plan type, and count of non-SUD comorbidities (0, 1, ≥2) documented on an inpatient or outpatient record during the study period based on Elixhauser comorbidity software refined for *ICD-10-CM* version 2021.1.

Total annual expenditures by service category (inpatient, outpatient, outpatient drugs) per enrollee for the study period were set to minimum $0 to eliminate artificial negative values (eg, payment reconciliation for services before the study period). Two-part regression models assessed enrollees’ expenditures by service category, controlling for all matching characteristics except for comorbidity count, which was replaced with indicators for each comorbidity (eg, hypertension) to address imbalanced proportions of SUD and control enrollees for specific comorbidities, plus indicators for enrollees’ SUD diagnoses by SUD type (eg, alcohol-related disorder). In the first part of the model, logistic regression assessed the probability of a greater than $0 total expenditure during the study period, and in the second part, generalized linear modeling assessed variation in enrollees’ total expenditure values relative to their SUD diagnosis status. The regression-adjusted annual attributable mean cost of SUDs per affected enrollee was the postestimation marginal effect point estimate of the models’ SUD diagnosis indicators when the 95% CI for the marginal effect point estimate was greater than 0. Two-part models accommodate observations (enrollees) with value of zero for the dependent variable (6.1% of analyzed enrollees had $0 total annual expenditure). The marginal effect point estimate was multiplied by the number of people in the US ESI population with SUD diagnoses from the weighted complete data source to mathematically model the total SUD medical cost.

## Results

Among 162 million 2018 ESI enrollees, 2.3 million (1.4%) had an SUD diagnosis (Table 2).^9^ The regression analysis sample included 210 225 individuals with an SUD diagnosis (121 357 [57.7%] male individuals; 68 325 [32.5%] aged 25-44 years) and 1 049 539 individuals with no SUD diagnosis (Table 3). This sample was used to model per-enrollee SUD costs, and the mean total annual expenditure was $26 051 (95% CI, $25 775-$26 327) among all enrollees with SUD diagnosis and $10 405 (95% CI, $10 331-$10 479) among all enrollees without SUD diagnosis. More than one-third (72 235 [34%]) of analysis sample enrollees with an SUD diagnosis had at least 2 comorbidities. The most common comorbidities among enrollees with an SUD diagnosis were depression (63 049 [30.0%]), hypertension (62 582 [29.8%]), and obesity (35 823 [17.0%]).

**Table 2.** Analysis Sample and Population Estimates, 2018^a^

Measure	Patients, No. (%)					
	From data source				US ESI population^d^	
	Per-enrollee cost analysis^b^		Total^c^			
	Enrollees (n = 14 602 603)	% With any SUD diagnosis (n = 210 225)	Enrollees (n = 26 158 337)	% With any SUD diagnosis (n = 367 106)	Enrollees (n = 162 136 077)	% With any SUD diagnosis (n = 2 258 062)
SUD diagnosis^e^	210 225 (1.4)	100.0	367 106 (1.4)	100.0	2 258 062 (1.4)	100.0
Alcohol	106 167 (0.7)	50.5	185 356 (0.7)	50.5	1 141 428 (0.7)	50.5
Cannabis	47 390 (0.3)	22.5	82 762 (0.3)	22.5	513 236 (0.3)	22.7
Hallucinogen	1593 (<0.1)	0.8	2725 (<0.1)	0.7	16 832 (<0.1)	0.7
Inhalant	632 (<0.1)	0.3	1151 (<0.1)	0.3	6889 (<0.1)	0.3
Opioids	56 070 (0.4)	26.7	97 834 (0.4)	26.7	611 396 (0.4)	27.1
Sedatives	13 172 (<0.1)	6.3	22 836 (<0.1)	6.2	138 954 (<0.1)	6.2
Stimulants	19 364 (0.1)	9.2	35 535 (0.1)	9.7	222 078 (0.1)	9.8
Other SUD	27 243 (0.2)	13.0	46 779 (0.2)	12.7	284 633 (0.2)	12.6

Abbreviations: ESI, employer-sponsored insurance; SUD, substance use disorder.

^a^ This table’s data are sourced from the 2018 Merative MarketScan Commercial Claims and Encounters databases.

^b^ Regression analysis sample limited to enrollees with continuous insurance enrollment during 2018 (≥1 d/mo) (7 608 070 of 26 158 337 [29.1%] excluded), mental health and SUD coverage (1 234 672 [4.7%] excluded), nonmissing key data (sex, age, region of residence, health plan type [eg, preferred provider organization; health maintenance and capitation plans excluded]; 2 712 992 [10.4%] excluded).

^c^ All enrollee records with weight values (approximately 97% of total enrollees).

^d^ Based on data source weights. Does not include approximately 15 million ESI enrollees with Medicare supplemental plans based on data source weighting in the Merative MarketScan Medicare Supplemental and Coordination of Benefits database. In combination, the Commercial Claims and Encounters and Medicare Supplemental and Coordination of Benefits databases indicate 178 million individuals in the ESI population in 2018, consistent with a Census estimate.^9^

^e^ Definitions appear in Table 1.

**Table 3.** Analysis Sample Description^a^

Measure	Enrollees with SUD diagnosis (n = 210 225)		Matched control enrollees (n = 1 049 539)	
	No. (%)	Total annual medical expenditure, mean (95% CI), $	No. (%)	Total annual medical expenditure, mean (95% CI), $
Total	210 225 (100)	26 051 (25 775-26 327)	1 049 539 (100)	10 405 (10 331-10 479)
Sex				
Male	121 357 (57.7)	24 396 (24 014-24 777)	605 269 (57.7)	9633 (9529-9737)
Female	88 868 (42.3)	28 311 (27 917-28 704)	444 270 (42.3)	11 457 (11 354-11 559)
Age, y				
0-17	10 954 (5.2)	21 875 (19 854-23 895)	54 719 (5.2)	7078 (6645-7511)
18-24	41 110 (19.6)	20 441 (19 981-20 899)	204 320 (19.5)	6263 (6109-6416)
25-44	68 325 (32.5)	21 567 (21 108-22 026)	341 330 (32.5)	8249 (8149-8348)
45-64	89 836 (42.7)	32 538 (32 104-32 971)	449 170 (42.8)	14 334 (14 206-14 461)
Residence region				
Northeast	49 769 (23.7)	27 176 (26 609-27 743)	248 619 (23.7)	10 669 (10 513-10 824)
North central	48 260 (23.0)	22 929 (22 470-23 387)	240 574 (22.9)	10 130 (9985-10 274)
South	82 297 (39.1)	25 973 (25 508-26 437)	411 306 (39.2)	10 218 (10 099-10 336)
West	29 899 (14.2)	29 433 (28 599-30 266)	149 040 (14.2)	10 926 (10 721-11 130)
Health plan type				
Comprehensive	10 885 (5.2)	23 507 (22 628-24 385)	54 014 (5.1)	10 560 (10 269-10 850)
EPO	1576 (0.7)	27 829 (24 945-30 713)	7800 (0.7)	11 949 (10 867-13 031)
POS	26 040 (12.4)	25 930 (25 213-26 646)	129 930 (12.4)	10 191 (9993-10 389)
PPO	125 925 (59.9)	26 659 (26 277-27 040)	629 224 (60.0)	10 709 (10 613-10 804)
CDHP	22 209 (10.6)	25 511 (24 709-26 312)	110 808 (10.6)	9836 (9579-10 093)
HDHP	23 590 (11.2)	24 505 (23 784-25 225)	117 763 (11.2)	9382 (9177-9587)
Non-SUD comorbidities, No.				
1	63 459 (30.2)	21 180 (20 682-21 677)	317 241 (30.2)	8027 (7939-8115)
≥2	72 235 (34.4)	46 215 (45 595-46 833)	359 643 (34.3)	20 616 (20 429-20 802)
Non-SUD comorbidities, type				
Depression	63 049 (30.0)	37 805 (37 244-38 367)	125 259 (11.9)	17 518 (17 240-17 795)
Hypertension	62 582 (29.8)	41 178 (40 491-41 864)	312 088 (29.7)	17 966 (17 776-18 157)
Obesity	35 823 (17.0)	36 645 (35 926-37 363)	269 336 (25.7)	14 072 (13 923-14 220)
Chronic pulmonary disease	24 818 (11.8)	44 488 (43 416-45 560)	122 569 (11.7)	17 746 (17 448-18 044)
Diabetes	17 822 (8.5)	53 957 (52 550-55 363)	119 519 (11.4)	23 096 (22 755-23 436)
Thyroid disorders	16 507 (7.9)	46 183 (44 881-47 485)	118 789 (11.3)	16 746 (16 463-17 027)
Arthropathies	11 842 (5.6)	53 551 (51 951-55 151)	45 340 (4.3)	27 339 (26 776-27 901)
Peripheral vascular disease	7791 (3.7)	74 960 (72 303-77 616)	35 541 (3.4)	35 535 (34 517-36 553)
Solid tumor without metastasis, malignant	3306 (1.6)	79 648 (75 700-83 595)	17 659 (1.7)	39 286 (38 006-40 564)
Metastatic cancer	1474 (0.7)	194 974 (185 309-204 638)	5215 (0.5)	135 223 (131 173-139 273)
Dementia	1055 (0.5)	83 383 (75 866-90 899)	1577 (0.2)	51 654 (46 262-57 046)
Cerebrovascular disease	945 (0.4)	105 206 (96 684-113 728)	2166 (0.2)	72 828 (67 734-77 921)
AIDS	876 (0.4)	61 997 (56 890-67 103)	2672 (0.3)	42 892 (40 630-45 153)
Valvular disease	730 (0.3)	102 134 (77 327-126 941)	5757 (0.5)	35 107 (32 795-37 419)
Solid tumor without metastasis, in situ	535 (0.3)	39 749 (34 164-45 333)	3624 (0.3)	17 861 (16 534-19 187)
Lymphoma	588 (0.3)	178 496 (159 389-197 603)	3374 (0.3)	96 222 (90 336-102 107)
Paralysis	578 (0.3)	126 875 (115 288-138 463)	1263 (0.1)	92 655 (84 963-100 347)
Kidney failure, severe	514 (0.2)	248 934 (219 887-277 980)	3121 (0.3)	147 478 (138 843-156 113)
Liver disease, moderate to severe	398 (0.2)	203 976 (179 577-228 375)	446 (<0.1)	105 525 (84 802-126 248)
Leukemia	312 (0.1)	208 070 (174 322-241 818)	1741 (0.2)	126 682 (113 963-139 401)
Congestive heart failure	38 (<0.1)	276 258 (178 480-374 036)	119 (<0.1)	378 986 (297 636-460 335)
Weight loss	<20	NR	30 (<0.1)	130 642 (47 226-214 059)

Abbreviations: CDHP, consumer-driven health plan; EPO, exclusive provider organization; HDHP, high deductible health plan; NR, not reported; POS, noncapitated point-of-service; PPO, preferred provider organization; SUD, substance use disorder.

^a^ Data were sourced from the 2018 Merative MarketScan Commercial Claims and Encounters databases. There were 1 to 5 control enrollees matched per enrollee with SUD diagnosis, creating modest differences in cohort proportions for some matching characteristics (sex, age, region of residence, health plan type, and count of non-SUD comorbidities).

Regression modeling that compared enrollees with SUD diagnoses and control participants estimated the annual attributable mean cost of any SUD diagnosis (including multiple) was $15 640 (95% CI, $15 340-$15 940) per affected enrollee, and the total estimated medical cost in the ESI population was $35.3 billion (Table 4). Among ESI enrollees with an SUD diagnosis, alcohol-related disorders ($10.2 billion) and opioid-related disorders ($7.3 billion) were the most expensive. Any SUD diagnosis (including multiple) was associated with nonzero attributable per-enrollee costs in all assessed service categories (inpatient: $6502 [95% CI, $6333-$6671]; outpatient: $8628 [95% CI, $8442-$8814]; outpatient drugs: $1503 [95% CI, $1412-$1594]). Those results appear to be influenced by the prevalence of alcohol-related disorders and opioid-related disorders; more than half of enrollees with an SUD diagnosis had an alcohol-related disorder and nearly 30% had an opioid-related disorder (Table 2). Each of those SUD types was associated with relatively high mean per–affected enrollee, per–SUD type inpatient ($3988 [95% CI, $3837-$4140] and $3570 [95% CI, $3336-$3804], respectively), outpatient (95% CI, $4875 [95% CI, $4716-$5033] and $6280 [95% CI, $5976-$6585]), and overall per affected enrollee ($8939 [95% CI, $8680-$9197] and $11 871 [95% CI, $11 317-$12 424]) costs (Table 4).

**Table 4.** Annual Medical Cost of SUDs in the US Employer-Sponsored Insurance Population, 2018^a^

Measure	Any SUD (n = 2 258 062)	Alcohol (n = 1 141 428)	Cannabis (n = 513 236)	Hallucinogen (n = 16 832)	Inhalant (n = 6889)	Opioids (n = 611 396)	Sedatives (n = 138 954)	Stimulants (n = 222 078)	Other (n = 284 633)
Per affected enrollee (95% CI), $^b^									
Total^c^	15 640 (15 340-15 940)	8939 (8680-9197)	9205 (8765-9645)	3029 (531-5528)	2658 (733-4582)	11 871 (11 317-12 424)	7120 (6266-7973)	5183 (4726-5639)	8041 (7269-8814)
Inpatient	6502 (6333-6671)	3988 (3837- 4140)	2934 (2729-3140)	1442 (771-2112)	1778 (305-3251)	3570 (3336-3804)	3874 (3497-4251)	2152 (1911-2393)	3456 (3152-3760)
Outpatient	8628 (8442-8814)	4875 (4716-5033)	5648 (5398-5897)	2056 (1363-2750)	2631 (1214-4048)	6280 (5976-6585)	3936 (3566-4305)	3653 (3359-3946)	4072 (3815-4329)
Outpatient drugs	1503 (1412-1594)	NS	NS	NS	NS	3318 (3125-3511)	795 (523-1068)	433 (217-649)	872 (624-1120)
Total, $ millions^d^	35 316	10 203	4724	51	18	7258	989	1151	2289

Abbreviations: NS, not significant; SUD, substance use disorder.

^a^ Costs are reported in 2018 US dollars. Data were sourced from the 2018 Merative MarketScan Commercial Claims and Encounters Databases.

^b^ Results are marginal effect estimates of SUD indicators from 2-part generalized linear regression models, as described in the Statistical Analysis section. Models controlled for SUD indicators (eg, alcohol-related diagnosis), sex, age, region of residence, health plan type, and non-SUD comorbidities, as listed in Table 3.

^c^ Total cost per affected enrollee was directly modeled (not a sum of inpatient, outpatient, and outpatient drugs results).

^d^ Total cost was the mathematical combination (product) of the statistically estimated per-affected enrollee cost point estimate and population size estimate when the 95% CI for the cost point estimate was statistically greater than 0. For example, total cost for alcohol-related disorders: $8939 × 1 141 428 = $10 203 million.

## Discussion

This study’s primary contribution is quantifying the annual medical cost of SUDs in the ESI population based on recent financial transactions. In assessing total annual expenditures (inpatient, outpatient, outpatient drugs) among ESI enrollees with SUD, this analysis expands what is known about attributable costs in the ESI population compared with recent research that used hospital discharge data to analyze only inpatient costs.^7^ The inpatient SUD cost estimates from the present study of the ESI population are higher than comparable inpatient SUD costs among patients with all health care payers in the previous study. This is primarily because modeling here addressed not just the cost difference that was associated with SUDs among patients treated in hospitals (eg, costs for inpatients with and without SUD diagnoses being treated for the same heart condition), but also the cost difference for the entire hospital encounter if associated with SUDs based on comparing individuals with the same demographic characteristics, health plan type, and comorbidities, but different SUD diagnosis status. The $35.3 billion SUD medical cost represents a small fraction of total US personal health care expenditures paid by private insurance ($1.1 trillion in 2018).^10^ However, in this study, 1% of the ESI population had an SUD diagnosis compared with 11% of workers who self-report SUD,^3^ suggesting the medical cost that employers and their health insurance payers face is likely far higher than reported here. Employers can take action by developing workplace supported prevention, treatment, and recovery programs.^11^

### Limitations

This study has limitations. Administrative records may inaccurately capture SUDs.^12^ Estimates reported here capture insurance-related expenditures (including out-of-pocket costs) among enrollees with SUD and mental health services coverage but not self-pay services. Treatment services for SUDs were not excluded (therefore, the cost estimates exceed the value of preventing SUDs before they occur) but are likely modest; only 12% of workers with SUDs report receiving treatment in the past year.^3^ Using exact matching to create a control cohort before regression modeling reduced the possibility of overestimating the attributable cost of SUD because the models compared enrollees who were substantially similar in terms of demographic, geographic, and insurance factors. However, it was not possible to exactly match enrollees by specific comorbidities, and some comorbidities were more or less common in the exposure cohort (eg, 30% of the analyzed SUD enrollees had a depression diagnosis during the analysis year compared with 12% of the control cohort, and 17% of the analyzed SUD enrollees had an obesity diagnosis compared with 26% of the control cohort). The regression models addressed this by controlling for specific comorbidities in the analysis of enrollees’ total annual expenditures. The regression analysis sample was a large convenience sample rather than a weighted ESI population analysis. A weighted regression analysis was not possible because there are no data source weighting factors for some enrollee exclusion criteria necessary for cost modeling (eg, health plan type). The estimated cost of any SUD diagnosis ($15 640 per affected enrollee and $35.3 billion in the ESI population) includes people with multiple SUD diagnoses and may be more meaningful than individual SUD type results; survey data suggest 40% of workers with illicit drug use disorders had comorbid alcohol use disorder.^5^ Additional research to determine how to cost-effectively implement SUD interventions for workplaces could benefit the ESI population.^13^ This study did not assess the cost of SUD among subgroups by demographic characteristics, which may obscure the presence of a greater cost burden among some groups.

## Conclusions

In this economic evaluation, we found that the estimated annual medical cost of SUDs in the non–Medicare eligible US ESI population was $15 640 per affected enrollee and $35.3 billion in total during 2018. The cost of strategies to support employees and their health insurance dependents to prevent and treat SUDs can be considered in terms of potentially offsetting the existing high medical cost of SUDs. Medical expenditures for SUDS represent the minimum direct cost that employers and health insurers face because not all people with SUDs have a diagnosis, and costs related to absenteeism, presenteeism, job retention, and mortality are not addressed.

## Notes

Supplement.
Data Sharing Statement
