# Supplementary material for: Medical Costs of Substance Use Disorders in the US Employer-Sponsored Insurance Population
Source: JAMA Netw Open. 2023 Jan 24;6(1):e2252378. doi: 10.1001/jamanetworkopen.2022.52378 (PMC9972180; doi:10.1001/jamanetworkopen.2022.52378)
Supplement: Supplement. — Data Sharing Statement [file jamanetwopen-e2252378-s001.pdf]

Data Sharing Statement

Li. Medical Costs of Substance Use Disorders in the US Employer-Sponsored Insurance Population. *JAMA Netw Open*. Published January 24, 2023.
doi:10.1001/jamanetworkopen.2022.52378

Data
Data available: No

Additional Information
Explanation for why data not available: Data sharing: Data source is publicly available.
